# Influence of post-thaw culture on the developmental potential of human frozen embryos

**DOI:** 10.1007/s10815-012-9793-z

**Published:** 2012-05-22

**Authors:** Mafalda L. Rato, António Gouveia-Oliveira, Carlos E. Plancha

**Affiliations:** 1 Centro Médico de Assistência à Reprodução—CEMEARE, Rua Alfredo Mesquita, 2E, 1600-922 Lisbon, Portugal; 2 Departamento de Bioestatística, Faculdade de Ciências Médicas, Universidade Nova de Lisboa, Campo dos Mártires da Pátria 130, 1169-056 Lisbon, Portugal; 3 Unidade de Biologia da Reprodução, Instituto de Histologia e Biologia do Desenvolvimento, Av. Professor Egas Moniz, 1649-028 Lisbon, Portugal

**Keywords:** Embryo cryopreservation, Blastomere proliferation, Culture environment, Implantation rate, Developmental competence

## Abstract

**Purpose:**

Apart from freezing/thawing related cryodamage, several additional factors have been identified as major players in the reduction of success rates after frozen embryo transfers. The post-thaw culture is particularly relevant as it may amplify environmental influences over a stressed embryo. In the present study the influence of the post-thaw culture duration on the implantation and developmental potential of cleavage stage embryos was evaluated.

**Methods:**

In this retrospective evaluation, that spanned an 8-year period, 631 frozen-thawed embryos were allocated to one of two study groups, depending on their post-thaw culture period: *1)* the long (18–24 h), or *2)* the short (2–5 h) culture group. Groups were compared regarding implantation rate and live birth rate *per* embryo transferred. This comparison was corrected for the most common confounding factors such as maternal age at oocyte pick-up, number of transferred embryos, developmental day at freezing, blastomere survival after thawing, catheter used for transfer and year of procedure.

**Results:**

Implantation and live birth rate *per* embryo transferred were inversely related to the duration of the post-thaw culture, as diminishing this period significantly increased both rates. Moreover, no advantage could be found for a long post-thaw culture period, even for embryos with observed mitotic activity.

**Conclusion:**

This retrospective analysis indicates that a short post-thaw culture period is associated with higher implantation and live birth rates *per* embryo. This study supports selection of frozen-thawed embryos strictly based on blastomere cryosurvival and raises the hypothesis that environmental factors may have an important role on embryo implantation and developmental potential during post-thaw culture.

## Introduction

Currently, the two most common procedures to select embryos for frozen embryo transfer (FET) differ in the duration of the post-thaw culture: one relies upon the observation of blastomere proliferation, requiring a longer culture, generally overnight; the other relies upon the observation of blastomere survival after thawing, requiring a shorter culture. The effectiveness of one strategy over another is still not clear as few studies have compared large series of embryos over a long time period. Additionally, all published results are influenced by biasing factors relating to the population demographics and to events described as modulators of FET outcome [2, 5, 6, 14, 18, 20, 22].

Although FET is generally considered a well established technique, a large number of studies concentrate on the reasons for its lower success rate when compared to fresh embryo transfers [1, 2]. Major contributors to such divergence have been extensively discussed and, as an attempt to elevate frozen embryo transfer (FET) outcome, the post-thaw assessment of mitotic resumption became a widely employed strategy for embryo grading and selection [5, 6, 14, 18, 20, 22]. As a consequence, the specific effect of post-thaw culture duration has remained neglected by most FET studies. In our centre, until 2005, post-thaw embryo selection was preferentially based on mitotic resumption. However, FETs were most frequently non-elective and embryos surviving the thawing process were generally the same embryos selected for transfer the following day, irrespective of subsequent mitotic resumption. Interestingly, after an internal evaluation of our FET program, a tendency towards a better outcome following transfer of embryos thawed on the same day as transfer was identified. In light of such observation, thawing began to preferentially be performed on the same day as transfer and embryo post-thaw selection based on mitotic activity was no longer a priority.

The current understanding that available embryo culture systems, although aiming to mimic the physiological environment, do not replace it completely [8], supported the modification of our post-thaw culture protocol. It additionally raised the hypothesis that frozen-thawed embryos may be particularly sensible to sub-optimal environmental influences during post-thaw culture, possibly interfering with potential to implant and development to a live birth. In order to explore the specific effect of post-thaw culture duration upon implantation and live birth rates, we conducted a retrospective evaluation spanning an 8-year period. Specifically, we compared the influence of two distinct post-thaw culture lengths, 2–5 h versus 18–24 h, on embryo implantation and full term developmental potential using a statistical approach designed to control for the influence of common biasing factors.

## Materials and methods

### Embryo inclusion and study period

This retrospective study evaluated all frozen-thawed embryos derived from FET cycles initiated at Centro Médico de Assistência à Reprodução (CEMEARE) in Lisbon, Portugal, during an 8-year period. FETs included in this study resulted from the cryopreservation of supernumerary embryos generated in the course of IVF/ICSI cycles. Irrespective of the number of FET cycles derived from each ovarian pick-up (OPU), only embryos derived from the first FET were eligible for the study. Only viable embryos were considered—defined as having retained ≥50 % of their initial number of blastomeres intact upon thawing.

Concerning the time span of the study, two main periods reflecting two different post-thaw methodologies were traversed. The first period extended mainly from May 2001 to June 2005 and coincided with the established tendency to perform an overnight post-thaw culture and select embryos for transfer based on their mitotic activity. The second period extended mainly from July 2005 to December 2008 and significantly represented our new tendency to reduce the culture period between thaw and transfer, relying only on post-thaw survival for embryo selection.

### IVF/ICSI procedures

Patients undergoing IVF/ICSI cycles were subjected to controlled ovarian stimulation using a GnRH agonist (Decapetyl, Ipsen Pharma Biotech, Signes, France) or antagonist (Orgalutran, Organon/Schering-Plough, UK; or Cetrotide, Serono, London, UK) for hypolalamic down-regulation; human menopausal (Menopur, Ferring GmbH, Kiel, Germany) or recombinant FSH (Gonal-F, Serono, UK; or Puregon, Organon/Schering-Plough, UK) for ovarian stimulation; and hCG (Pregnyl, Organon/Schering-Plough, Oss, Holand; or Ovitrelle, Serono, London, UK) for induction of oocyte maturation. After ovarian puncture COCs were incubated in IVF medium (all culture media from MediCult/Origio, Jyllinge, Denmark). Zygotes and day 2 embryos were cultured in ISM1 medium and transferred to ISM2 medium at day 3. Cleaving embryos were scored every day for blastomere number, cleavage plane and degree of fragmentation and graded accordingly. Higher grade embryos were selected for fresh embryo transfer. Surplus embryos presenting an adequate relation between blastomere number and developmental day, a correct cleavage plan and a maximum of 20 % fragmentation were cryopreserved.

### Cleavage stage embryo slow freezing and rapid thawing

The freezing-thawing procedures and media remained unchanged during the complete study period. According to the slow freezing protocol, embryos were sequentially equilibrated at room temperature in a HEPES-buffered medium containing cryoprotectants up to a final concentration of 1.5 M 1,2-propanediol (PROH) and 0.1 M sucrose (Medicult/Origio embryo freezing pack). Embryos were loaded into ministraws (CryoBioSystems, France) and cooled using a programmable freezer (MiniCool, AirLiquid, France). The cooling program started at 20°C and ran as follows: temperature was taken to −7°C at a rate of 2°C/min and manual seeding was performed by touching the extremes of the straw with a nitrogen-cooled forceps. From that point on, the temperature was slowly decreased at 0.3°C/min to −30°C. A final and very fast drop to −150°C was accomplished decreasing the temperature at a rate of 50°C/min. Straws were then plunged into liquid nitrogen and stored until thawing.

Rapid thawing was achieved by quickly placing the straws on hand for a minimum of 30 s. Embryos were then discharged to a 4-well dish and washed from the cryoprotectants at room temperature, using sequential thawing media (Medicult/Origio embryo thawing pack). According to their developmental day upon freezing, embryos were placed in appropriate medium, checked for blastomere survival under an inverted microscope and incubated until transfer. Long post-thaw incubation consisted of an overnight culture for an 18–24 h period before transfer. Short post-thaw incubation consisted of a culture for a 2–5 h period before transfer.

### Uterine transfer of frozen-thawed embryos

Embryo replacement to the uterus was performed following endometrium preparation using Leucoprolide (Decapetyl depot 0,1 mg/ml, Ipsen Pharma Biotech, Signes, France)—a GnRH agonist, for hypothalamic suppression and 17β-estradiol (Estrophem, Novo NordisK, Novo Allé, Denmark) in order to mimic the natural occurring estrogens until the endometrium reached 8 mm thick and the serum estradiol levels reached ≥150 mg/dl. From this stage on, progesterone (Utrogestan, Jaba Recordati, Sintra, Portugal) was added to the previous therapeutic scheme for endometrium support and embryo transfer was scheduled

**Fig. 1:**
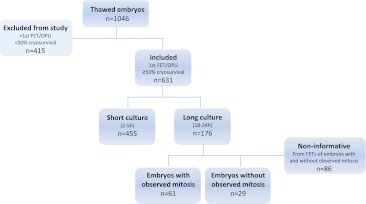
Flow chart summarizing thawed embryo accountability of the study. Implantation rates and live birth rates *per* embryo were compared between the long and the short culture groups. These outcomes were additionally compared between embryos with observed mitotic resumption from the long culture group and embryos without observed mitosis both in the long and short culture groups

Before FET, embryos were rechecked for viability, placed in pre-equilibrated Universal Transfer Medium (UTM) and loaded into an appropriate catheter (Frydman or TDT, Laboratoire CCD, Paris; or Sydney IVF embryo transfer set, Cook OB/GYN, USA). Regarding mitotic activity, three types of FETs occurred: FETs in which all embryos transferred had cleaved during the culture period (with observed mitosis); FETs in which none of the embryos transferred had cleaved during the culture period (without observed mitosis); and FETs in which some of the embryos had and some had not cleaved during the culture period. In this last group of FETs, embryos could not be assigned to either group and were considered non-informative regarding mitotic resumption. In the short culture group, mitotic activity was not assessed due to biological inadequacy of such observation (Fig. 1).

Clinical pregnancy was defined by the presence of a gestational sac with a fetal heartbeat on ultrasound examination at 6 weeks of gestation.

### Statistical analysis

The primary efficacy endpoints were the implantation rate (IR—the number of gestational sacs *per* number of embryos transferred) and the live birth rate *per* embryo (LBR/E—the number of newborns *per* number of embryos transferred). Secondary efficacy endpoints were pregnancy, multiple pregnancy, gestational sac involution and delivery rates. In addition, we looked into the relevance of observing mitotic activity to implantation rate and the live birth rate *per* embryo.

Differences between groups were tested with multiple linear regression for interval-measured endpoints and logistic regression for binary endpoints. Whenever endpoints referred to episodes, analyses were clustered by subject and robust “Sandwich” (Huber-White) standard errors were used to account for non-independence within subjects. In order to control for systematic differences between groups, all analyses were adjusted for the following potential confounding factors: maternal age at OPU, number of embryos transferred, day of embryonic development at freezing, blastomere cryosurvival after thawing, catheter used for transfer and procedure year. All tests are two-tailed. Results are reported as adjusted odds-ratios (AOR) with 95 % confidence intervals (95 % CI). Differences were considered significant if *p* < 0.05. Statistical analysis was performed using STATA 11 (Stata Corporation, College Station, Texas). Whenever appropriate, writing and analysis followed the STROBE [21].

## Results

Between May 2001 and December 2008, 631 viable embryos out of a total of 1046 were analyzed, 176 in the long culture group (18–24 h of post-thaw culture) and 455 in the short culture group (2–5 h of post-thaw culture). Particularly, the embryos analyzed derived from the first FET of 265 OPUs. Considering the culture groups, 70 FETs were included in the long and 195 in the short culture group. As expected in a retrospective study, there were differences between the study groups regarding demographic, clinical and embryological characteristics of the populations (Table 1). In order to account for their influence on the results, all analyses were adjusted for mean maternal age at OPU, number of transferred embryos, developmental day at freezing, catheter used for transfer and year of procedure.

**Table 1:** Demographic, clinical and embryological characteristics of FETs in the two different post-thaw culture groups

	Short culture group (2–5 h)	Long culture group (18–24 h)	p
Maternal age at OPU, mean (SD)	33.5 (4.5)	32.6 (4.2)	0.03
Developmental day at freezing, n (%)			<0.001
Day2	92 (41.1)	60 (75.0)	
Day3	132 (58.9)	20 (25.0)	
Post-thaw surviving embryos, %	71.4 (534/748)	67.8 (202/298)	0.26
No. Transferred embryos, mean (SD)	2.3 (0.7)	2.5 (0.8)	0.036
Catheter used for transfer, n (%)			0.017
Frydman	60 (27.3)	24 (30.8)	
Cook	66 (30.0)	11 (14.1)	
TDT	94 (42.7)	43 (55.1)	
Year of procedure, n (%)			<0.001
2001–2004	43 (19.2)	60 (75.0)	
2005–2008	181 (80.8)	20 (25.0)	

**Fig. 2:**
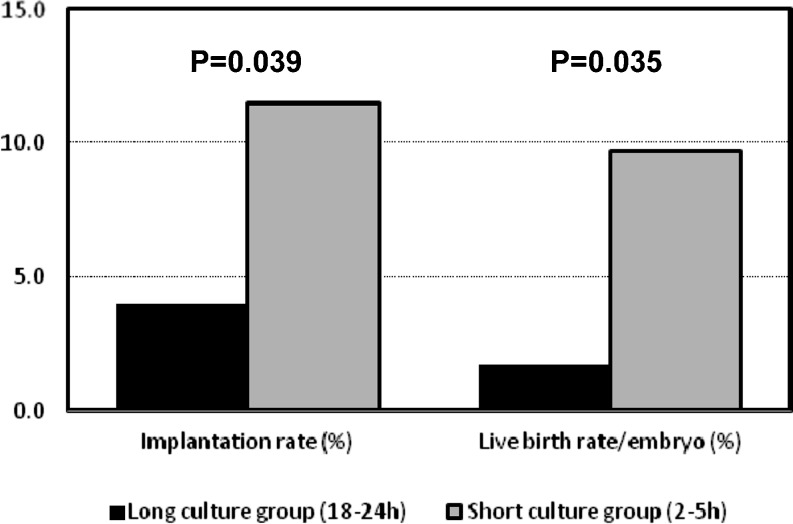
Influence of distinct post-thaw culture periods. Embryos cultured for a long (18–24 h) or a short (2–5 h) period exhibit very distinct outcomes: the unadjusted probability of frozen embryos to implant and develop to term significantly increases if briefly cultured

Both implantation rate and live birth rate *per* embryo transferred were found to be significantly higher in the short than in the long culture group (IR: 11.5 % *vs* 4.0 %; LBR/E: 9.7 % *vs* 1.7 %) (Fig. 2). The adjusted odds-ratio for implantation rate in the short to the long culture group was 2.32 (95 % confidence interval 1.04 to 5.17, *p* = 0.04) and for live birth rate was 2.96 (95 % confidence interval 1.08 to 8.15, *p* = 0.04) (Table 2).

**Table 2:** Outcome of FETs following distinct post-thaw culture periods in the short to the long culture group

	Adjusted odds-ratio	95 % CI	p
Implantation rate	2.32	1.04; 5.17	0.039
Live birth rate *per* embryo	2.96	1.08; 8.15	0.035
Pregnancy rate	2.32	0.89; 6.02	0.085
Multiple pregnancy rate	2.32	0.89; 6.02	0.085
Gestational sac involution rate	1.17	0.20: 6.81	0.857
Delivery rate	2.98	0.97; 9.17	0.057

**Fig. 3:**
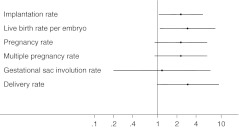
Adjusted odds-ratios and 95 % confidence intervals of the study variables in the short to the long culture group. Six potential confounding factors were controlled: maternal age at oocyte pick-up, number of embryos transferred, day of embryonic development at freezing, blastomere cryosurvival after thawing, catheter used for transfer and year of procedure

Regarding the secondary efficacy variables (Table 2 and Fig. 3), although the observed differences were in favor of the short culture group in the pregnancy rate (21.4 % *vs* 12.5 %, AOR 2.32, 95 % CI 0.89 to 6.02), they did not reach statistical significance (*p* = 0.085). Differences were also not statistically different either in the rate of multiple pregnancies (*p* = 0.085), neither in the gestational sac involution rate (*p* = 0.86).

In order to assess the relationship between blastomere mitotic activity and embryo developmental potential, we compared the outcome of embryos transferred after observed mitotic resumption in the long culture group (61 embryos) and embryos transferred after a short post-thaw culture (in which no mitotic activity was observed, 455 embryos). These groups were not statistically different in the mean number of embryos transferred (2.2 ± 0.7 and 2.3 ± 0.7; *p* = 0.43) and mean maternal age at OPU (32.0 ± 4.6 and 33.5 ± 4.4; *p* = 0.06). No statistical advantage was found from transferring embryos cultured for a long post-thaw period and with proven mitotic division, over embryos cultured for a reduced length of time regarding implantation rate (odds ratio 1.99, *p* = 0.20) and live birth rate *per* embryo (odds ratio 1.97, *p* = 0.26). Additionally, using only data from the long culture group, we also compared the developmental potential of embryos with proven (*n* = 61) or not observed (*n* = 29) blastomere mitotic activity, since there was a similar mean number of embryos transferred (2.4 ± 0.9, *p* = 0.43) and a mean maternal age at OPU (33.9 ± 3.9, *p* = 0.20). Again, no statistically significant difference was found in implantation rate (odds ratio 1.88, *p* = 0.71). The numbers were however insufficient to compare the live birth rate *per* embryo (Table 3).

**Table 3:** Relevance of observing mitotic activity. Comparison of the outcomes of embryos transferred after observed mitotic resumption in the long culture group (*n* = 61) to embryos without mitotic activity in the short and in the long culture groups

Group	Adjusted odds-ratio	95 % CI	p
Short culture (*n* = 455)			
Implantation rate	1.99	0.70; 5.63	0.20
Live birth rate per embryo	1.97	0.60; 6.41	0.26
Long culture (*n* = 29)			
Implantation rate	1.88	0.07; 49.40	0.71
Live birth rate per embryo	n.a.		

Cleavage stage embryos were allocated to different transfer groups according to mitotic resumption: long culture group, where mitotic resumption was observed in all embryos transferred; long culture group, where no mitotic resumption was observed in all embryos transferred; short culture group, where no mitotic resumption was expected to occur during the culture period

## Discussion

The results of this retrospective study indicate that a short post-thaw culture period is associated with higher implantation and live birth rate *per* embryo. To our knowledge, this is the first study to identify a long post-thaw culture period as being responsible for loss of embryo implantation and developmental potential. Additionally, this study reinforces the advantages of a frozen-thawed embryo selection approach strictly based on blastomere cryosurvival [3, 19].

### The observed differences between study groups rely on a strong statistical plan and are independent of common biasing factors

Although the conclusions from this study are based on retrospective data and have the inherent limitations of retrospective studies, there are strengths in its design that afford strong confidence on the results. First, the study was based on a large sample of transferred embryos spanning a period of 8 years. Second, the quality of data is high, without missing data or a single case lost to follow up. Third, to overcome the lack of randomization between groups, the analysis accounted for a number of possible confounding factors of FET outcomes such as maternal age at OPU, number of embryos transferred, day of embryonic development at freezing, blastomere cryosurvival after thawing, catheter used for transfer and year of procedure. The latter two variables are often neglected but importantly reflect increasing operator (embryologist and clinician) experience and the continuous improvement of equipments and devices, such as embryo transfer catheters. Particularly, adjusting the analysis for the year of procedure was essential as the superiority of one culture duration (long or short), could be biased by the preferential association with a time period (from 2001 to 2004 and from 2005 to 2008). In addition, the analysis accounted for the clustering of episodes (transfers) within subjects (couples) and adjusted standard errors for possible non-independence of episodes within subjects. Therefore, conclusions arising from this work are adequately supported by the statistical analysis plan. Finally, although pregnancy and live birth rates are commonly used outcomes, the first is still an intermediate measure and does not discriminate between implantation of one or more embryos, while the second returns a result in terms of oocyte pick-up or embryo transfer procedures and not in relation to each transferred embryo. As this study aims to center on individual embryo developmental potential following two different post-thaw culture durations and not on live birth *per* woman, implantation rate and live birth rate *per* embryo were considered to be more accurate and informative outcome measures. In fact, more important than the actual, unadjusted, values observed for implantation and live birth rate *per* embryo represented in Fig. 2 are the adjusted differences between groups (Fig. 3), which estimate the independent effect of distinct post-thaw culture periods after controlling for six potential confounding factors.

### Shorter post-thaw culture periods allow embryos to maintain higher implantation and developmental potential

Our results indicate that the probability of frozen-thawed embryos to implant and develop to term is significantly increased if embryos are only briefly cultured after thawing. This observation highlights the environmental impact that post-thaw culture length has on embryo developmental potential. Negative influences of culture upon embryo development have been pointed out and, although embryo culture aims to mimic the fallopian tube and uterine environment, it always implies induction of some related stress [8–10, 17]. It thus seems reasonable to propose that frozen-thawed embryos may be less able to cope with, and adapt to, sub-optimal environments such as the currently available culture systems [16]. By reducing the post-thaw culture duration, there will be a decrease of culture-related stress, better preserving embryonic developmental potential.

As previously reported, many factors have been pointed out as influencing post-thaw outcome, but their relative contributions are variably accepted [11]. When comparing ART outcomes, maternal age at OPU and number of embryos transferred are usually accepted as the two most important causes of bias in intra- and inter-assay comparisons. At freezing, the two most referred embryological factors are embryo grading [2, 7] and embryo developmental day [13, 15], while after thawing, the two most discussed embryological factors are blastomere cryosurvival [5, 19] and the visualization of blastomere mitotic resumption [20, 22]. In the light of the present study, we suggest that “post-thaw culture duration” should be considered as another confounding factor, strongly influencing developmental potential and consequently FET outcome.

### Is it useful to prolong culture after thawing in order to observe initial blastomere proliferation?

Although some previous reports seemed to demonstrate that post-thawed cleaving embryos with observed blastomere proliferation hold a higher developmental potential than non-cleaved ones [14, 20, 22], we found no evidence of increased implantation and live birth rates *per* embryo after transferring only embryos where blastomere proliferation had been observed after a long post-thaw culture. These results contrast with others reported for the same type of transfers, possibly due to the statistical analysis or to the non-elective approach of our FETs [20] and to the marked influence of common biasing factors, such as age and number of embryos transferred, in the most recently reported data [6]. Finally, the negative influence that a prolonged post-thaw culture exerts on embryonic developmental potential reinforces the concept that the inability to develop in vitro is no evidence of developmental incompetence in vivo [4, 12]. Our results are in favor of such concept, as the outcome of post-thaw cleaved embryos and those selected based only upon survival is similar. This indicates that the developmental potential of non-cleaving embryos could be better preserved if they would only be briefly exposed to post-thaw in vitro conditions.

### Final considerations and conclusion

We all agree that the best outcome achieved after embryo cryopreservation will result from embryos with a high score before freezing, that not only retain their initial blastomere number, but above all retain their developmental competence and that must end up resuming mitotic activity in the uterine cavity. Our results suggest that a prolonged post-thaw culture period can be causing a decrease in the implantation and developmental potential of thawed cleavage stage embryos. The present study raises the interesting proposal that it may not be desirable to actually see the resumption of mitotic activity in some blastomeres of thawed embryos, because that usually requires a long post-thaw culture period. This hypothesis now demands further validation through prospective randomized studies.

## References

[1] Andersen AN, Goossens V, Bhattacharya S, Ferraretti AP, Kupka MS, Mouzon J (2009). Assisted reproductive technology and intrauterine inseminations in Europe, 2005: results generated from European registers by ESHRE. Hum Reprod.

[2] Edgar DH, Bourne H, Speirs AL, McBain JC (2000). A quantitative analysis of the impact of cryopreservation on the implantation potential of human early cleavage stage embryos. Hum Reprod.

[3] El-Toukhy T, Khalaf Y, Al-Darazi K, Andritsos V, Taylor A, Braude P (2003). Effect of blastomere loss on the outcome of frozen embryo replacement cycles. Fertil Steril.

[4] Griffiths TA, Murdoch AP, Herbert M (2000). Embryonic development in vitro is compromised by the ICSI procedure. Hum Reprod.

[5] Guerif F, Bidault R, Cadoret V, Couet ML, Lansac J, Royere D (2002). Parameters guiding selection of best embryos for transfer after cryopreservation: a reappraisal. Hum Reprod.

[6] Joshi BV, Banker MR, Patel PM, Shah PB (2010). Transfer of human frozen-thawed embryos with further cleavage during culture increases pregnancy rates. J Hum Reprod Sci.

[7] Karlstrom PO, Bergh T, Forsberg AS, Sandkvist U, Wikland M (1997). Prognostic factors for the success rate of embryo freezing. Hum Reprod.

[8] Lane M, Gardner DK (2005). Understanding cellular disruptions during early embryo development that perturb viability and fetal development. Reprod Fertil Dev.

[9] Leese HJ, Sturmey RG, Baumann CG, McEvoy TG (2007). Embryo viability and metabolism: obeying the quiet rules. Hum Reprod.

[10] Leese HJ, Baumann CJ, Brison DR, McEvoy TG, Sturmey RG (2008). Metabolism of the viable mammalian embryo: quietness revisited. Hum Reprod.

[11] Mandelbaum J, Junca AM, Plachot M, Alnot MO, Alvarez S, Debache C (1987). Human embryo cryopreservation, extrinsic and intrinsic parameters of success. Hum Reprod.

[12] Plachot M, Belaisch-Allart J, Mayenga JM, Chouraqui A, Tesquier L, Serkine AM (2002). Outcome of conventional IVF and ICSI on sibling oocytes in mild male factor infertility. Hum Reprod.

[13] Salumets A, Tuuri T, Maèkinen S, Vilska S, Husu L, Tainio R (2003). Effect of developmental stage of embryo at freezing on pregnancy outcome of frozen-thawed embryo transfer. Hum Reprod.

[14] Salumets A, Suikkari AM, Mäkinen S, Karro H, Roos A, Tuuri T (2006). Frozen embryo transfers: implications of clinical and embryological factors on the pregnancy outcome. Hum Reprod.

[15] Sifer C, Sellami A, Poncelet C, Martin-Pont B, Porcher R, Hugues J (2006). Day 3 compared with day 2 cryopreservation does not affect embryo survival but improves the outcome of frozen-thawed embryo transfers. Fertil Steril.

[16] Stokes PJ, Hawkhead JA, Fawthrop RK, Picton HM, Sharma V, Leese HJ (2007). Metabolism of human embryos following cryopreservation: implications for the safety and selection of embryos for transfer in clinical IVF. Hum Reprod.

[17] Summers MC, Biggers JD (2003). Chemically defined media and the culture of mammalian Preimplantation embryos: historical perspective and current issues. Hum Reprod Up.

[18] Tang R, Catt J, Howlett D (2006). Towards defining parameters for a successful single embryo transfer in frozen cycles. Hum Reprod.

[19] Van den Abbeel E, Camus M, Van Waesberghe L, Devroey P, Van Steirteghem AC (1997). Viability of partially damaged human embryos after cryopreservation. Hum Reprod.

[20] Van der Elst J, Van den Abbeel E, Vitrier S, Camus M, Devroey P, Van Steirteghem AC (1997). Selective transfer of cryopreserved human embryos with further cleavage after thawing increases delivery and implantation rates. Hum Reprod.

[21] von Elm E, Altman DG, Egger M, Pocock SJ, Gotzsche PC, Vandenbroucke JP (2007). The Strengthening the Reporting of Observational Studies in Epidemiology (STROBE) statement: guidelines for reporting observational studies. Lancet.

[22] Ziebe S, Bech B, Petersen K, Mikkelsen AL, Gabrielsen A, Andersen AN (1998). Resumption of mitosis during post-thaw culture: a key parameter in selecting the right embryos for transfer. Hum Reprod.

